# Challenges for psychiatric nurses working with non-suicidal self-injury adolescents: a qualitative study

**DOI:** 10.1186/s12912-023-01542-z

**Published:** 2023-10-13

**Authors:** Xuting Li, Shiyan Liu, Yusheng Tian, Juan He, Hui Chen, Meng Ning, Zengyu Chen, Jiaxin Yang, Yamin Li, Jiansong Zhou

**Affiliations:** 1 Clinical Nursing Teaching and Research Section, The Second Xiangya Hospital, Central South University, Changsha, Hunan China; 2 Department of Psychiatry, National Clinical Research Center for Mental Disorders, The Second Xiangya Hospital, Central South University, Changsha, China; 3 Department of Thoracic Surgery, The Second Xiangya Hospital, Central South University, Changsha, Hunan China; 4 Xiangya School of Nursing, Central South University, Changsha, Hunan China

**Keywords:** Non-suicidal self-injury, Adolescents, Psychiatric nurses, Qualitative study

## Abstract

**Background:**

Psychiatric nurses play a crucial role in treating and supporting adolescents with non-suicidal self-injury (NSSI) in China. However, few studies have explored their experiences and challenges.

**Objectives:**

The aim of this qualitative study was to describe the challenges experienced by psychiatric nurses when working with adolescents having NSSI behaviors.

**Methods:**

This was a descriptive qualitative study using phenomenological approach. 18 psychiatric nurses from psychiatric wards were recruited from a tertiary hospital from Changsha, Hunan province, China. In-depth interview was performed for each participant collecting information about their feelings and experiences taking care of NSSI adolescents. ATLAS.ti 8 was used to enter data and perform thematic analysis following the six-phased process described by Braun and Clarke.

**Results:**

Two main themes and five sub-themes were summarized in this study. Nurses experienced both (1) Internal challenges (Lacking knowledge and skills to deal with NSSI adolescents and Feeling hard and stressful working with NSSI adolescents) and (2) External barriers (Unrealistic high expectations from family and schools, Uncooperative parents and Little help from communities and schools).

**Conclusions:**

Psychiatric nurses had to face with their own negative feelings, insufficient knowledge and skills, alongside with pressures and little help from family, schools and communities when working with NSSI adolescents. Targeted training programs of treating NSSI adolescents and their supporting systems be performed in nurses, furthermore, family, schools and societies should also be raised.

## Background

Non-suicidal self-injury (NSSI) is defined as direct and deliberate destruction of one’ body tissue in ways that are not socially or culturally sanctioned and without suicidal intention [1]. In 2013, DSM-5 recognized NSSI as a distinct clinical phenomenon and made a call for more systematic research by including Non-Suicidal Self-Injury-Disorder (NSSI-D) as a condition requiring further research [2]. The aggregate 12-month prevalence of NSSI in children and adolescents worldwide is 19.5% (95% CI: 13.3–27.6%) [3]. In China, it’s estimated that the prevalence of NSSI in middle school students aged 13 ~ 18 years is 27.4% [4], higher than the global average rate. Meanwhile, due to the stress, social restrictions and isolation cause by COVID-19 pandemic in recent years, the number of adolescents with NSSI were increasing and had raised great concerns [5]. Non-suicidal self-injury have a great impact on personal health and is correlated with an increased risk of suicidal ideation and behaviors [6]. Studies showed that there was a ten times greater risk of suicide behavior among students with NSSI than others without NSSI, and the risk was even higher for recurrent NSSI [7]. Studies also reported a notable increase of suicide deaths among outpatients and inpatients with history of self-injury [8]. In this case, effective treatment and management of NSSI behaviors in adolescents could be an important step in preventing suicide [9].

Till now, there are no specific psychopharmacological treatment for NSSI, medications such as fluoxetine and aripiprazole are only used for treatment the comorbid mental disorders [10]. Thus, psychotherapies performed by medical staff are crucial treatment for adolescents with NSSI. Systematic review reported 6 psychotherapeutic interventions focused on reducing adolescents’ NSSI [11], including Developmental Group Psychotherapy (DGP), Therapeutic Assessment (TA), Cutting Down Program (CDP), Emotional Regulation Individual Therapy for Adolescents (ERITA), Treatment for Self-Injurious Behaviors (T-SIB) and Intensive Contextual Treatment (ICT). Most of these interventions were designed based on Cognitive-Behavioral Therapy (CBT), Dialectical Behavioral Therapy (DBT), Functional Family Therapies (FFT), and special trainings of clinicians were needed. In China, with the relatively insufficient psychiatrists nationwide [12], the implementation of psychotherapies very much relies on other mental health professors, such as trained nurses. Psychiatric nurses in China often spend most of their time with patients in the wards, trained and certified nurses will also conduct psychotherapies for adolescents with NSSI.

However, treating and managing adolescents’ NSSI behaviors is a complex and systematic work. It’s not only a psychological disease that may co-occur with many mental disorders, but also a sophisticated social issue. Among lifetime NSSI patients, 59.6% meet the criteria for at least one mental disorder [13], while others without any psychiatric disorder are also more likely to be diagnosed with one [14]. Meanwhile, some adolescents viewed NSSI as a normal and effective way to regulate emotions, and they didn’t want to seek help or raise attention [15]. The complicated and heterogenous condition of NSSI adolescents make it hard for psychiatric nurses to manage. Besides, nurses also need to work with parents and school teachers over adolescents’ NSSI behaviors, because family conflict, poor relationships with caregivers and school bullying were all important risk factors for NSSI in adolescents [16, 17]. Although mental health nurses showed more confidence and positive attitudes compared with other nurses [18], they are still facing with various challenges, which may influence the effectiveness of treatment by hindering creation of meaningful therapeutic relationships. Fully understanding those challenges could identify areas of support relevant for both nurses and other stakeholders, aiming to improve patient care.

Previous researchers investigating nurses’ experiences of working with individuals who self-harm tended to focus on adults [19], and challenges referred as being emotionally affected by self-harm, managing professional boundaries and environmental expectations. As for working with adolescents’ NSSI behaviors, quantitative studies showed that it’s easy for psychiatric nurses to feel emotionally affected, with powerlessness and uncertainty as two most prevalent negative emotions [20]. Qualitative research showed that, community mental health nurses confronted with personal and interpersonal conflicts when working with adolescents who self-harm within the context of working with systems surrounding the adolescents [21]. However, the above studies were mostly conducted in developed countries, and few studies focused on psychiatric nurses in China.

In China, there are only 0.34 psychiatric nurses per bed in top-tier psychiatric hospitals, and 6.8 psychiatric nurses per 100,000 population [12]. With higher rate of NSSI behaviors in Chinese adolescents (12.9%~27.4%) [4] and the shortage of psychiatrists in China, many NSSI adolescents are admitted to hospitals for treatment. One qualitative study interviewing parents and medical staff in a Chinese hospital showed that staff perceived adolescent patients with suicide-related behaviors as difficult to engage, and parents were also not satisfied with the existing hospital services [22]. Lack of human resources in hospital and systematic treatment models of NSSI were the major challenges [22]. Psychiatric nurses are the mental health professionals who spend most of the time with adolescent patients, yet few studies reported their special obstacles and experiences.

Therefore, this study aims to explore the internal and external challenges psychiatric nurses experienced working with NSSI adolescents through a descriptive qualitative approach. The study will help us better understand the obstacles of clinical practice faced by psychiatric nurses in the context of Chinese society and provide evidence for improving the situation. Hopefully, this could lead to better management of adolescents’ NSSI in the future.

## Methods

### Design

This was a qualitative study aiming to describe challenges for psychiatric nurses working with non-suicidal self-injury adolescents in China.

### Setting and sample

The setting of this study was a tertiary hospital from Changsha, Hunan. Tertiary hospitals are defined as institutions with more than 500 beds in China, and are the major provider of inpatient service in Chinese health-care system [23]. Located in the central China, Changsha is the capital city of Hunan, with a population of 10.04 million and the Growth Domestic Product ranking 15th nationwide [24]. According to the China’s seventh national population census in 2020 [25], people aged 0 ~ 14 years old and 15 ~ 59 years old accounted for 16.64% and 68.03% of the whole population in Changsha respectively. With over 200 middle schools and high schools in Changsha, the average education duration of people aged over 15 years old was 11.52 years [25]. In general, it’s a typical and representative second-tier city in China.

Purposive sampling was used to recruit participants. The research samples were psychiatric nurses working with NSSI adolescents. The inclusion criteria incorporated the following: (1) registered nurses currently working in psychiatric wards; (2) having experience of working with adolescents’ NSSI behaviors; (3) the ability to complete the interview with normal listening and communicating abilities; (4) agreeing to participant with informed consent. The exclusion criteria were as follows: (1) withdrawing at any time; (2) unable to communicate verbally; (3) having been diagnosed with severe mental illness or cognitive impairment. According to literature [26], to achieve maximum sampling variation, nurses with different gender, age, education background and working experiences were all invited. With authorization of nurse managers in the hospital and wards, phone number of all psychiatric nurses were acquired. Then, nurses who meet the inclusion and exclusion criteria were purposively invited through phone calls and texts. Afterwards, researchers would meet those who showed interest to participant and get their formal written informed consent. With informed consent, nurses were officially included in the study.

Finally, 18 nurses (15 females, 3 males) agreed to participate. The mean age of the participants was 31.92 years (SD = 6.82, range: 23 yrs. − 46 yrs.), and the average duration of professional practice was 9.15 years (SD = 6.95; range: 0.5 yrs. − 20 yrs.). Details of the participants were shown in Table 1.

**Table 1:** Demographics of participants (N = 18)

Characteristics	N (%)	Mean ± SD
**Education**		
Associate college	1 (5.5%)	
Bachelor’s degree	16 (89.0%)	
Master’s degree	1 (5.5%)	
**Gender**		
Male	3 (16.7%)	
Female	15 (83.3%)	
**Age (years old)**		31.92 ± 6.82
**Duration of professional practice (years)**		9.15 ± 6.95

### Data collection

Face-to-face in-depth interviews with participants were conducted from April 2022 to May 2022. All participants were invited in a psychotherapy room within the ward and the whole interview process lasted 30 ~ 45 min. Semi-structured questions of the interview guide were developed based on the aims of the investigation and the research gaps identified from the literature review, including (1) How do you feel when working with NSSI adolescents? Why? (2) What’s the most difficult part? Why? (3) How does working with NSSI adolescents affects you? Why? (4) What could be done to improve the situation? The interviews were conducted in Mandarin. Data saturation was reached when no new analytical information aroused anymore.

### Data management and analysis

Data analysis began concurrently with the first interview. Two researchers (Researcher A and B) conducted the interviews and transcribed recording verbatim into texts within 24 h afterwards. Researcher A was a postgraduate student majoring in nursing. Researcher B was a psychiatric nurse with master degree of nursing, who have worked in psychiatric wards for years. Both of them have learned the qualitative approach during their master education, and have performed qualitative interviews before. ATLAS.ti 8 was used for further analysis. Thematic analysis was used, following the six-phased process described by Braun and Clarke [27]. It is a rigorous and flexible method that can be used to identify, analyze, organize, describe and report themes found within a data set across a range of epistemologies and research questions. A completed phases included reading and re-reading interview transcripts to familiarize with raw data, generating initial codes by attaching labels to identified interesting aspects in the data, and searching for themes by sorting related codes hierarchically. The raw themes and subthemes were presented and reviewed by research team, who possess clinical psychiatry background and qualitative research experience, until the scope and content of each theme could be clearly and succinctly described and consensus was reached.

### Ethical considerations

This study was approved by the Ethical Committee of National Clinical Medical Research Center at the hospital (No.2022-010). Formal written informed consent was obtained from each participant. Apart from the aim and procedure of this study, nurses were also told that the participation wouldn’t affect them or their career, the whole interview would be audio-recorded and the anonymous records would only be used for this study. Besides, they were told about their rights to refuse to answer any question or withdraw at any time as well. With agreement to participant, they would sign an informed consent, after which they would be officially included in the study and interviewed. All methods were performed in accordance with the guidelines and regulations of the Declaration of Helsinki.

## Results

**Fig. 1:**
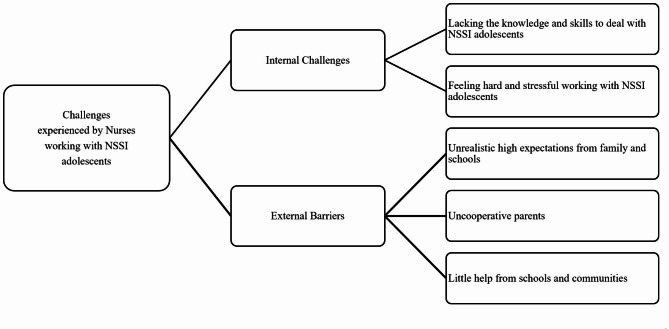
Thematic map of two major themes (middle blocks) and five sub-themes (right blocks)

Two major themes were extracted from the analysis: [1] internal challenges and [2] external barriers. The internal challenges were described as difficulties from nurses themselves while the external barriers were obstacles form family and schools. Within each theme, we also identified several sub-themes. Figure 1 showed the two sub-themes of the internal challenges and three sub-themes of the external barriers. The following is a summary of the results, including samples of corresponding responses from participants.

### Theme 1: internal challenges

#### Sub-theme 1: lacking the knowledge and skills to deal with NSSI adolescents

Most participants felt they were unprepared when dealing with NSSI adolescents, especially lacking the knowledge and skills to establish reliable relationship with adolescents and provide professional help. Schools of nursing didn’t provide such lessons and they had to learn from their seniors through daily work. There were few systematic trainings for nurses before they get involved with NSSI patients, even though they were the ones NSSI adolescents spent most of the time with in psychiatric wards.

*In fact, we (nurses) are on the way. But we didn’t learn (these knowledge) in schools… we didn’t learn (NSSI intervention) systematically. (P9)*

*There lacks adequate relevant vocational training before work started. (P6)*

#### Sub-theme 2: feeling hard and stressful working with NSSI adolescents

Stressful and hard were common negative emotions nurses experienced after dealing with NSSI adolescents, and these feelings may last for a rather long time and affect their own mental health. Witnessing the scars caused by repeated self-harm, listening to miserable feelings of NSSI adolescents and dealing with exhausted parents were main causes of negative emotional responses, and the situation may get worse if the nurses were too young to possess crucial skills and knowledge.

*It is hard, and sometimes stressful, particularly for rookies with no practical experience…the mental health of nurses also needs to be concerned. (P17)*

### Theme 2: external barriers

#### Sub-theme 1: unrealistic high expectations from family and schools

Some participants complained about the unrealistic high expectations from family and schools. Parents tended to think it could be completely cured once the doctors diagnosed their children and the responsibilities were soon transferred to psychiatrists and nurses. Meanwhile, schools also wanted the certification or guarantee from hospitals that the adolescents were fully recovered and ready to go to school, which was actually unrealistic. With family and schools imposing the duties on hospitals, nurses were overwhelmed and felt more difficult dealing with adolescents’ NSSI behaviors.

*Um, because in the hospital, parents had laid all their hope on doctors. They often said “You should help me solve this problem immediately and perfectly.’ And schools asked for phone calls from the hospital to guarantee that the student was recovered and ready to go to school. (P7)*

*He (patient) generally thought this problematic behavior (NSSI) could be cured because the doctor had given him a medical diagnosis and would only discharge him after evaluation. (P3)*

#### Sub-theme 2: uncooperative parents

Many adolescents developed NSSI behaviors due to their problematic parents, who were not being very cooperative during psychotherapies. Parents often didn’t realize their problems and failed to make changes. In this case, when adolescents were discharged from the hospital, they were back in the same family environment that may cause their initial NSSI behaviors, in the meantime, the parents, however, holding the idea that their kids were cured, they would fail to continue the psychotherapies for the kids. With parents failed to cooperative, it’s easy for adolescents to harm themselves repeatedly. It’s frustrated for nurses when NSSI reoccurred constantly.

*After discharge, as a nurse, even if I can keep in touch with them, not every patient and their family are willing to continue to cooperate…In their concept, discharge is cure. (P15)*

*Both the parents and family members had no self-control. They don’t know how to control their emotion and feelings toward the kids. (P10)*

*In Chinese culture, many parents believe that harsh parenting was the only way to educate kids and sometimes they may use physical violence. With this misunderstanding and lack of psychological knowledge, many parents failed to realize their problems and change the strict parenting style. (P17)*

#### Sub-theme 3: little help from communities and schools

Since managing adolescents’ NSSI was a long-term work, nurses would need social support outside the hospital. However, they received little help with few long-term management programs in communities and schools concerning NSSI or self-harm in adolescents. Many schools had hired psychological teachers, but they rarely paid attention on NSSI and its influential factors such as peer bullying. As for communities, there were systematic programs of chronic disease, while little was concerned about mental health. Besides, both schools and communities expected psychological professors from hospitals to perform follow-up assessments, so they normally failed to realize their responsibilities. These situations resulted in extra follow-up work for nurses outside the hospital and increased the difficulties when facing adolescents with repeated NSSI due to same adverse stimulations from schools.

*Schools had few knowledge about this (psychological health and NSSI) …. I doubted that whether there were real education classes about psychological health in classrooms? … Support is not enough… (P8)*.

*Teachers should receive more trainings on communication skills (with NSSI adolescents) …Besides, the study pressures and peer influences, for example, the experience of peer bullying, should be noticed. (P16)*

*Last year, the school hired a Ph.D. in psychology to give them extra classes. However, it seemed that he did not mention the topic of suicide in the class. What he said was to teach parents not being so anxious when educating their children. (P5)*

*Most communities have monitoring programs for chronic diseases… but there still lack such services for mental diseases. (P13)*

*Psychiatric hospitals were demanded (by communities) to undertake all the follow-up assessments with insufficient medial resources. (P9)*

## Discussion

To our knowledge, this is the first study describing challenges experienced by psychiatric nurses working with NSSI adolescents in China. Results of this study showed that they would face both internal challenges (Lacking knowledge and skills to deal with NSSI adolescents and Feeling hard and stressful working with NSSI adolescents) and external barriers (Unrealistic high expectations from family and schools, Uncooperative parents and Little help from communities and schools).

Psychiatric nurses were the majority of mental health professionals in China, understanding what difficulties they faced was rather important. According to data from the China Health Statistical Yearbook 2020, there were 3 times more psychiatric nurses (6.8 per 100,000) than psychiatrists (2.9 per 100,000) in 2019 [12]. In this case, nurses took charge of many clinical works, including taking care of NSSI adolescents’ daily life in hospital, giving medications according to doctors’ prescription, educating adolescents and their parents with self-care knowledge. For those with qualifications, they also had to help psychiatrists organizing psychological therapies and perform counselling for both inpatients and outpatients. Meanwhile, study showed that the general population tended to choose a healthcare institution (usually tertiary hospitals) or specialty psychiatric hospitals without being referral by the primary care psychiatrists, resulting in over-crowding of tertiary hospitals [28]. Therefore, nurses from tertiary hospitals were the mental health professionals NSSI adolescents would spend most of the time with in China, and their work would affect outcomes of adolescents greatly. However, our study revealed that nurses would encounter many challenges working with NSSI adolescents, which were not mentioned in previous studies.

Nurses reported that they lacked knowledge and skills to deal with NSSI adolescents and wanted more comprehensive and professional trainings urgently. Our results support the previous studies concerning working with self-harm adolescents in community [21]. But unlike former studies in which these feelings may gradually go way in their daily practice with supervision and support [21], nurses in our study expressed confuse and low confidence due to lack of sufficient abilities no matter how long they have been working in this area. One possible reason may be that there is indeed no specific psychopharmacological treatment for NSSI, and updated new interventions require lots of trainings. Another reason may lie in the education system of psychiatric nurses in China. The majority of nurses didn’t receive much lessons about psychological disorders until they worked, let alone suicide and self-harm. They had to learn through clinical practice, which was both inefficient and incomplete. Even though hospitals had regular training programs on mental health care, there were few concerning NSSI behaviors in adolescents. A nationwide study showed that there were only 3.3% child units in top-tier psychiatric hospitals [29], indicating the mental health of children and adolescents hadn’t raised enough attention. This situation led to difficult work for nurses and unqualified treatment for adolescents. Specific training regarding understanding and managing self-harm could benefit nurses working with those adolescents [30]. Considering the reality of clinical work, we assume that peer-learning and case-based learning could be effective ways for psychiatric nurses gaining knowledge and skills of NSSI. Based on the sociocultural theory, learning collaboratively with peer workers through interpersonal communication and feedbacks is the best approach in the context of actual clinical practice [31]. Nevertheless, those programs should be organized systematically centralized in the psychological therapies and management of adolescents with NSSI. Besides, the establishment of child and adolescent units in hospitals may also promote psychiatric nurses setting their professional area in caring for NSSI adolescents.

Meanwhile, negative emotions such as hard and stressful was another challenge nurses had to face when working with NSSI adolescents. Seeing the scars directly and unprepared to provide specific mental health care could worsen the situation, as our participants mentioned that young nurses seemed to experience more fear, stressful and helplessness. This was in consistent with previous studies reporting the negative emotions referring as powerlessness, frustration and conflicts [21]. Psychiatric nurses were required to be patient, calm, discriminative and empathic for NSSI adolescents, which were described as “double-edged sword” towards themselves [21]. Therefore, mindfulness-based programs and increased reflective practice could benefit psychiatric nurses in detection of their own emotions, consideration of their contributions in relationships with NSSI adolescents and further setting clear boundaries to protect themselves [19, 32].

Apart from internal challenges, psychiatric nurses had experienced resistance and little support from family and schools in China. Both parents and school teachers tended to view the discharge as cured. But the chronic interpersonal stress caused by parents, classmates and teachers has reciprocal associations with NSSI in adolescents [33]. Irresponsibility of families and schools resulted from their lack of knowledge and awareness of adolescent NSSI. Parents were hard to recognize NSSI as an adverse action which would need professional psychological assistances. Their ignorance, shame and stereotype attitude towards NSSI behaviors led to reluctance in cooperation with nurses. While nurses were complaining about parents’ difficulties to engage, parents were also not satisfied with the hospitalization treatment and advices. In context of Chinese culture, influential factors of adolescents’ NSSI included sex-bias discrimination, overly high expectations from parents and inappropriate parenting style [34]. Without an improved family environment provided by parents, adolescents would have to spend much more effort to recover and be more easily to repeat self-harm. In this case, education for parents and family psychological therapies were important. Meanwhile, the stigma of NSSI from schools exist either implicitly or explicitly also result in irresponsibility in helping NSSI adolescent adapting. Negative peer relations could positively related to Chinese adolescent NSSI [35], for example, the growing rate experience of bullying in the decades concurred with the increase in adolescent NSSI [16]. Aware of this situation years ago, Chinese government had taken actions in protecting psychological health of students in schools, by publishing policies about mental health education in schools, requiring at least two psychological teachers in each school, forbidding extra homework or lessons outside schools and etc. However, participants in our study showed that there was still a long way to go before schools developing a perfect system for adolescents with NSSI or other psychological disorders. NSSI behaviors increase in adolescence with a decline in young adulthood [36], which means that dealing with supporting systems around NSSI adolescents is necessary and vital for mental health nurses. This is more inevitable in China, with few mental health professionals in communities. In this case, we also recommend trainings of systemic working for psychiatric nurses, such as attachment-based family therapy [37], to make them feel more confident and sophisticated during this process.

Moreover, communities provided little help during this process with lacking long-term management of NSSI adolescents, which also increased the workload of psychiatric nurses in hospitals. With the shortage of psychiatrists and psychiatric nurses nationwide [12], there were few mental health professionals in communities to perform continuously monitoring and managing adolescents with NSSI. And there was a misunderstanding that once NSSI adolescents were discharged, they were cured and no longer needed to be taken care of specially, and nurses should be responsible for the follow check-ups. Haunted by these unrealistic expectations, psychiatric nurses expressed dissatisfactions with both communities and schools. This situation may get better with the development of nurse practitioners in China. Psychiatric mental health nurse practitioners in American were reported to make a significant contribution to behavioral healthcare delivery, especially in public mental health settings [38]. This indicated the promising future for psychiatric nurses, who could be trained systematically to be psychiatric nurse practitioners with prescription rights. Hopefully, the psychiatric nurse practitioners in China would be the main psychological professionals providing mental health care in primary heath institutions, which could relieve the burden of tertiary hospitals and be able to take good care of NSSI adolescents outside hospitals.

Our study revealed the internal and external challenges faced by Chinese psychiatric nurses working with NSSI adolescents in a qualitative approach, which could provide innovative perspectives and understandings towards the current situation of psychological care for self-harm adolescents in China. And valuable suggestions could be made through this study. Firstly, for psychiatric nurses, systematic trainings are in urgent need to help them prepared to work with NSSI adolescents and their surrounding systems. Regular mental health evaluation, self-emotion regulation practice and targeted intervention should also be performed for their psychological health. Secondly, as for parents, necessary lessons targeting communication with adolescents and establishment of harmonious family environment should be given, while they also need to learn how to take care of their children outside hospitals. Thirdly, school teachers should master the knowledge and skills to recognize peer bullying and early signs of psychological disorders, to prevent suicide or self-harm behaviors in schools. Last but not least, efficient long-term management of NSSI adolescent should be established in communities.

This study also had several limitations. Firstly, the participants of this study all worked in the same hospital. Since the catchment area in which the nurses work is the same, the types of patients may not differ very much. Considering that NSSI adolescents may have various morbidities, multi-centered studies would be needed to make up to this limitation. Moreover, nurses from other mental health services (territorial, school, etc.) may also provide different viewpoints. Secondly, as one of the interviewers was a colleague from the same practices, this may influence participants expression of opinions through self-presentation and impression management strategies or group-think effects. Thirdly, to maximize the sample variation, we have included nurses with a variety of working experiences from different wards. The different support and training tools among wards and high standard deviation of working duration may cause some bias. In this case, hierarchical group interviews of different groups of nurses could help explain. Finally, the distribution of gender is unbalanced in the study as 15 out of 18 participants are females. More opinions from male aspect should also be included as they may perceive different experiences.

## Conclusions

This study reported internal and external challenges psychiatric nurses faced when dealing with NSSI adolescents. Internal challenges refer as lacking sufficient knowledge and skills, and experiencing negative emotions. In the meantime, high expectations and passive cooperation from family, little help from schools and communities increased extra external pressure on taking care of NSSI adolescents. We suggested that not only should targeted training programs of treating NSSI adolescents and their supporting systems be performed in nurses, in-depth awareness and understanding of NSSI among adolescents, family, schools and societies should also be raised. Establishing self-harm and suicidality management system including hospital, schools and communities could be helpful. In addition, nurses may need to practice reflective thinking and self-care skills to protect their own mental health.

## Data Availability

The datasets generated and/or analysed during the current study are not publicly available due [individual privacy could be compromised] but are available from the corresponding author on reasonable request.

## Abbreviations

NSSI: non-suicidal self-injury
DGP: Developmental Group Psychotherapy
TA: Therapeutic Assessment
CDP: Cutting Down Program
ERITA: Emotional Regulation Individual Therapy for Adolescents
T-SIB: Treatment for Self-Injurious Behaviors
ICT: Intensive Contextual Treatment
CBT: Cognitive-Behavioral Therapy
DBT: Dialectical Behavioral Therapy
FFT: Functional Family Therapies
